# Laboratory capacity of Greek hospitals for diagnosis of salmonellosis and surveillance systems’ performance in the years of economic crisis, 2010–2016

**DOI:** 10.1017/S0950268818002686

**Published:** 2018-09-28

**Authors:** K. Mellou, E. Saranti-Papasaranti, G. Mandilara, T. Georgakopoulou

**Affiliations:** 1 Hellenic Centre for Disease Control and Prevention, Athens, Greece; 2 National Reference Centre for Salmonella, National School of Public Health, Central Public Health Laboratory, Hellenic Centre for Disease Control and Prevention, Vari, Attica, Greece

**Keywords:** Laboratory capacity, notification rate, salmonellosis, surveillance system, underreporting

## Abstract

Austerity might have affected the capacity of public hospitals in Greece to diagnose salmonellosis (laboratory capacity) over the period 2010–2016, as well as the performance of the existing surveillance systems. The scope of this paper is to present data on laboratory capacity over these years, as well as the results of a two-source capture-recapture study (data from Mandatory Notification System and National Reference Laboratory System for *Salmonella*). The main findings were that: (a) laboratory capacity was high and steady besides the financial crisis, (b) the estimated number of laboratory-confirmed cases (*n* = 6017, 95% CI 5892–6142) resulted in an incidence rate (7.9 cases/100 000 population) almost twice than that reported by the two systems Mandatory Notification System (MNS); 4.1 and National Reference Laboratory System (NRLS); 4.5 cases/100 000 population, (c) underreporting was high for both systems (MNS; 47.5% and NRLS; 42.8%) and (d) differences by geographical region, size and type of hospital were identified. We suggest that (a) specific interventions are needed to increase completeness of the systems by type of hospital and geographical region, (b) record linkage can help in estimating the disease burden in a more valid way than each system separately and (c) a common electronic database in order to feed one system to the other could significantly increase completeness of both systems.

## Introduction

Salmonellosis is a foodborne disease caused by *Salmonella* spp. that results in a high global morbidity and mortality. The World Health Organization estimated that non-typhoidal *Salmonella* spp. caused 78 million cases of foodborne illness, 28 693 deaths and 2 183 146 disability-adjusted life years globally in 2010 [1]. According to the European Food Safety Authority and the European Centre for Disease Prevention and Control, the EU/EEA annual salmonellosis notification rate for confirmed cases during the period 2010–2016 ranged from 20.3 to 21.9 per 100 000 population [2–4].

In Greece, there are two parallel surveillance systems for salmonellosis, both paper-based; Mandatory Notification System (MNS) for clinical cases and surveillance through the National Reference Laboratory System for *Salmonella* (NRLS), which is voluntary, but universal. Based on the MNS data, the mean annual notification rate of salmonellosis for the period 2010–2016 was 4.1 cases per 100 000 population (s.d. 1.37), having an increasing trend after 2014 [5].

There are limitations associated with the use of data from these surveillance systems if the degree of underreporting is not addressed [6]. Quantification of underreporting is needed in order to estimate the actual burden of the disease in the country, detect outbreaks early and evaluate policies for improving food safety [7, 8]. Since 2010 several efforts have been made to improve disease notification by medical doctors (in terms of routine feedback to hospitals regarding the management of cases/outbreaks of salmonellosis, publications emphasising the importance of notification, etc.) [9, 10].

On the other hand, during the same period, the country was in the middle of a financial crisis that began in November 2009 but deepened after the implementation of austerity measures in early 2010.

According to the Organization for Economic Co-operation and Development (OECD), health care spending in Greece has been consistently decreasing over the last years (total spending per capita in 2010 was USD 2696 and in 2016 was USD 2223), following the financial crisis [11]. The health care system, which is a highly centralised mixed model, incorporating both tax-based financing and social health insurance has experienced the consequences [12]. Strong annual growth increases were reversed after 2009 and there have been significant cuts in health spending (5.4% for the period 2003–2009 *vs.* −5.0% for the period 2009–2016) [11, 13]. Still, the national health system provides universe coverage to the population. Greek residents including uninsured people, legal and illegal migrants can visit the emergency rooms of the public hospitals, free of charge.

In Greece, the spending to GDP ratio has fluctuated, approaching close to 10% in 2010, before returning at around 8% of GDP in the following years [11]. In 2016, the USA spent more than eight percentage points above the OECD average (9.0% of Gross Domestic Product (GDP)) on health [11, 14]. A group of ten high-income OECD countries, including Switzerland, Germany, Sweden, France, Japan and Canada followed with about 11% of GDP going to health services [11, 14, 15]. Studies have demonstrated that service quality of public hospitals in Greece deteriorated during this period and that the decrease of the health care spending has resulted in increased workload for medical staff [16–20]. Additionally, the financial crisis might have also affected the capacity of the Greek hospitals to diagnose salmonellosis cases (perform cultures).

The objective of this paper is to: (a) investigate whether there have been significant changes in the laboratory capacity of Greek hospitals to diagnose salmonellosis over the period 2010–2016, (b) estimate the actual number of laboratory-confirmed salmonellosis cases at Greek hospitals during the same period, (c) assess underreporting of salmonellosis to both existing surveillance systems, (d) investigate whether there have been significant changes of underreporting rate over the years and (e) identify factors related to underreporting rate in each one of the surveillance systems in order to propose appropriate strategies for the improvement of the systems’ completeness.

## Methods

### Laboratory capacity

Since 2010 the Department of Epidemiological Surveillance and Intervention of Hellenic Centre for Disease Control and Prevention (HCDCP) collects data on the capacity of the microbiological laboratories of the public hospitals of the country to diagnose foodborne diseases. Each year, in January an official letter is sent to the administration of hospitals requesting for information on the current capacity of the hospital's laboratory to perform testing for a series of pathogens, including *Salmonella* spp. Hospitals are asked to complete a structured form and sent it back to HCDCP within 3 weeks. The official letter accompanying the request for data explains that one of the benefits from this recording is to have evidence on important changes of laboratory capacity compared with previous years and be able to assess to some degree the under-ascertainment rate of foodborne diseases in the country, which is an important element, for assessing the diseases’ burden. Additionally, changes of ‘laboratory capacity’ of the hospitals are directly linked to changes of the recorded notification rate for reasons other than an actual change of the morbidity.

After the 3 weeks time, the information is usually requested again from the hospitals that have not yet replied and an extension is given for the collection of data. Data are recorded in a specially designed database, are analysed and the report is sent to relevant stakeholders (directors of hospitals, Ministry of Health, etc.). Usually, the aforementioned work is finalised by the end of March each year.

The overall laboratory capacity of public hospitals to diagnose salmonellosis is calculated by dividing the number of hospitals that routinely perform cultures for *Salmonella* spp. by the total number of hospitals for which the information is available.

### Capture-recapture study

The two-source capture-recapture method was employed on the basis of salmonellosis notifications from NRLS and MNS [21, 22]. Clinical doctors notify new salmonellosis cases to local public health authorities and HCDCP through the MNS. In specific, after the laboratory confirmation of the case (isolation of *Salmonella* spp. from stool, urine, body site (e.g. infected wound) or any normally sterile body fluids and tissues (e.g. blood, CSF, bone, synovial fluid, etc.)) doctors complete the respective notification form of MNS that contains the name and demographic characteristics of the cases (sex, date of birth, place of residence), clinical symptoms, date of notification and laboratory data. Reported cases are classified in accordance with the European case definition and only cases with at least one of the following four; diarrhoea, fever, abdominal pain, vomiting are recorded [5].

In parallel with the notification of cases from clinical doctors, microbiologists are requested to send *Salmonella* spp. isolates to the national reference center located in Attica region for further typing (NRLS system). Each isolate is accompanied by a short form that includes the name and demographic characteristics (name, age, sex, date of birth, region) of the patient and the date of specimen collection. Isolates are serotyped and results are sent to the microbiological laboratories of the hospitals. Further molecular typing is also performed, in the case of clusters/outbreaks.

The purposes of the two systems are common but not identical. Both systems aim at monitoring the temporal distribution of salmonellosis and early detection of outbreaks, however, an additional aim of NRLS is to follow the trends of specific serotypes, identify emerging serotypes and proceed to molecular techniques for the identification of open outbreaks.

Information from the two sources was merged to estimate the real number of cases and the completeness of each source. All salmonellosis cases (non-typhoidal) reported during the period 2010–2016 at any of the two surveillance systems were included in the study. Data were checked for duplicates and in case there were two or more isolates from the same patient only the first one was included in the analysis. Isolates from asymptomatic cases were excluded from the database of NRLS.

A joint database was generated in order to include all reported cases of both systems. The detection of the same individuals in the two sources was determined by name, sex, age, date of notification and reporting hospital.

### Statistical analysis

The actual number of laboratory-confirmed cases (*N*) and the respective 95% confidence intervals (95% CI) were estimated with the use of (a) Chapman's formula and (b) Chao's lower bound estimator for a two-source capture-recapture data [23, 24].

Chapman's formula is traditionally used in capture-recapture studies with two sources [21]. However, the results of the analysis could be biased if the two data sources are positively dependent, thus generating underestimation of cases. Chao's lower bound estimator has been shown to be less affected by source dependence and provides more reliable estimates unless both sources are independent. Odds ratio (OR) would be close to unity when the two sources are independent [22]. In order to compute the OR, we estimated the number of individuals that did not appear in either source (*f*_00_) (Table 1) [23]. The estimate is given by the formula: 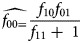. Underreporting of salmonellosis to each system was estimated by dividing the reported number of cases by the estimated number of laboratory-confirmed cases, as determined by capture-recapture analysis using both estimators. Underreporting rate of salmonellosis was also estimated by year and month of notification, type of hospital (public/private), hospital beds (<250/⩾251) and geographical region of the country (Attica/rest of Greece). Also, underreporting rates and these factors possibly affecting notification were compared between the two notification systems.

**Table 1.** The two-source situation

Source 1		1	0	
Source 2	1	*f*_11_	*f*_01_	*L*_2_
	0	*f*_10_	*f*_00_	
		*L*_1_		

The *t* test and Mann–Whitney *U* test were performed to compare the mean rates between two independent groups. Furthermore, a test of linear trend was performed, after computing underreporting rates for each year of follow up, by setting up regression analysis for testing the statistical significance of the seasonality [25].

The statistical analysis was performed using Microsoft Excel and STATA statistical software and *P*-values less than 0.05 were considered statistically significant.

### Ethics

The HCDCP is the competent authority for surveillance of communicable diseases according to Greek legislation and has been officially authorised to receive, treat and temporarily store personal data of infectious diseases cases by the Greek Authority for Personal Data Protection. Personal data were used only for the purposes of the matching procedure. All the necessary measures to protect the confidentiality of personal data were taken during the whole process. Access to the data was restricted to the personnel involved in data analysis and personal data were removed from the datasets after matching.

## Results

### Laboratory capacity

Based on the hospital records, as described in Table 2, the overall laboratory capacity of the Greek hospitals to perform cultures and diagnose salmonellosis was high (more than 80%), for the period 2010–2016.

**Table 2.** Capacity of microbiological laboratories of public hospitals to perform cultures for *Salmonella* spp., Greece, 2010–2016

	2010	2011	2012	2013	2014	2015	2016
Capacity to perform cultures for *Salmonella* spp.^a^	100/115 (87%)	102/116 (88%)	81/89 (91%)^b^	76/87 (87%)	67/83 (81%)	75/87 (86%)	82/89 (92%)

a Number of hospitals that routinely perform cultures for *Salmonella* spp. divided by the total number of hospitals for which the information was available.

b Drop of the number of hospitals after 2011 had mostly to do with the fact that some hospitals were closed or merged with others of the same area.

### Estimation of the actual number of cases and of underreporting rates

**Fig. 1.**
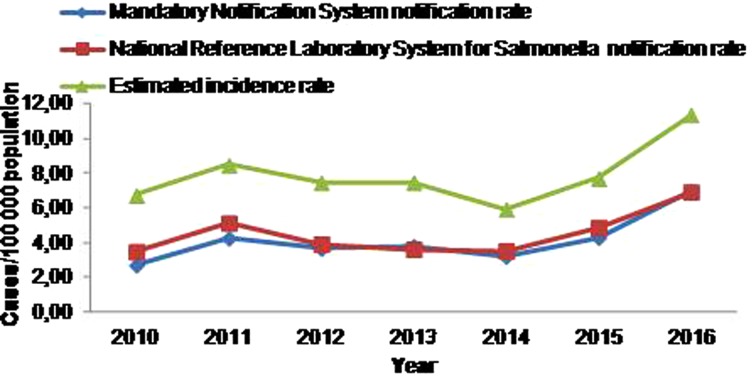
Time trend of salmonellosis notification rate at the Mandatory Notification System and the National Reference Laboratory System for *Salmonella* and estimated incidence rate, Greece, 2010–2016.

During the 7-year study period, 3161 salmonellosis cases were notified to MNS, 3444 cases to NRLS and 1809 cases were notified to both systems (Table 3), resulting to a mean annual notification rate of 4.1 and 4.5 cases per 100 000 population, respectively. The estimated actual number of laboratory-confirmed salmonellosis cases at Greek hospitals, for the period 2010–2016, was 6017 (95% CI 5892–6142), resulting to a mean annual incidence rate of 7.9 cases per 100 000 population. The salmonellosis notification rate and estimated incidence rate from the MNS and the NRLS for the period 2010–2016 is depicted in Figure 1.

**Table 3.** Estimated total number of salmonellosis cases and estimated underreporting of Mandatory Notification System and National Reference Laboratory System for *Salmonella*, by Chapman's and Chao's estimators, Greece, 2010–2016

				Total no. of estimated cases (Chapman's formula)			Total no. of estimated cases (Chao's formula)		
	No. records in MNS	No. records in NRLS	Matched records	Total cases (*N*) (95% CI)	MNS underreporting (%)	NRLS underreporting (%)	Total cases (*N*) (95% CI)	MNS underreporting (%)	NRLS underreporting (%)
All cases	3161	3444	1809	6017 (5892–6142)	47.5	42.8	6086 (5951–6221)	47.6	42.9
By year									
2010	301	386	155	748 (685–811)	59.8	48.4	761 (695–827)	60.5	49.3
2011	475	570	287	943 (895–991)	49.6	39.5	951 (902–1001)	50.1	40.1
2012	404	433	211	828 (773–883)	51.2	47.7	830 (775–886)	51.3	47.8
2013	419	397	202	822 (766–879)	49.1	51.7	824 (767–881)	49.2	51.8
2014	348	384	206	648 (610–686)	46.3	40.8	650 (611–689)	46.5	40.9
2015	467	528	294	838 (800–877)	44.3	37.0	842 (802–881)	44.5	37.3
2016	747	746	454	1227 (1183–1271)	39.1	39.2	1227 (1183–1272)	39.1	39.2
By type of hospital									
Public	2735	2620	1440	4975 (4857–5094)	45.0	47.3	4978 (4860–5097)	45.1	47.4
Private	419	796	369	904 (880–927)	53.6	11.9	1000 (960–1040)	58.1	20.4
By hospital beds									
<250	1310	1147	673	2232 (2157–2307)	41.3	48.6	2243 (2166–2319)	41.6	48.9
⩾251	1785	2057	1104	3325 (3243–3408)	46.3	38.1	3343 (3259–3426)	46.6	38.5
By region									
Attica	1263	2012	992	2561 (2509–2614)	50.7	21.4	2703 (2637–2769)	53.3	25.6
Rest of Greece	1891	1404	817	3249 (3140–3357)	41.8	56.8	3322 (3207–3437)	43.1	57.7
By month of notification									
January	120	123	57	258 (223–292)	53.4	52.3	259 (223–295)	53.7	52.5
February	101	103	55	188 (166–211)	46.4	45.3	189 (166–212)	46.6	45.5
March	107	113	65	186 (167–204)	42.3	39.1	186 (168–205)	42.5	39.3
April	153	182	100	278 (257–299)	45.0	34.5	281 (258–303)	45.5	35.1
May	210	220	126	366 (340–392)	42.7	39.9	367 (340–393)	42.8	40.0
June	282	288	173	469 (442–496)	39.9	38.6	470 (442–497)	39.9	38.7
July	405	448	246	737 (698–776)	45.0	39.2	739 (700–779)	45.2	39.4
August	562	661	359	1034 (991–1078)	45.7	36.1	1042 (997–1086)	46.0	36.5
September	465	523	278	874 (830–919)	46.8	40.2	878 (883–923)	47.0	40.4
October	344	365	182	689 (641–737)	50.1	47.0	690 (642–739)	50.2	47.1
November	261	271	122	578 (523–633)	54.9	53.1	580 (524–636)	55.0	53.3
December	151	134	46	436 (353–518)	65.3	69.2	441 (355–528)	65.8	69.6

As far as the estimated underreporting of the MNS and NRLS system is concerned, it was 47.5% and 42.8%, respectively, for salmonellosis cases. As shown in Table 3, estimates of cases were not very different using both formulae, since the two sources were independent (OR 1, 95% CI 0.9–1.1) and thus for convenience reasons only estimators as determined by Chapman's formula are included in the text.

### Underreporting rates over the years and seasonal trend

**Fig. 2.**
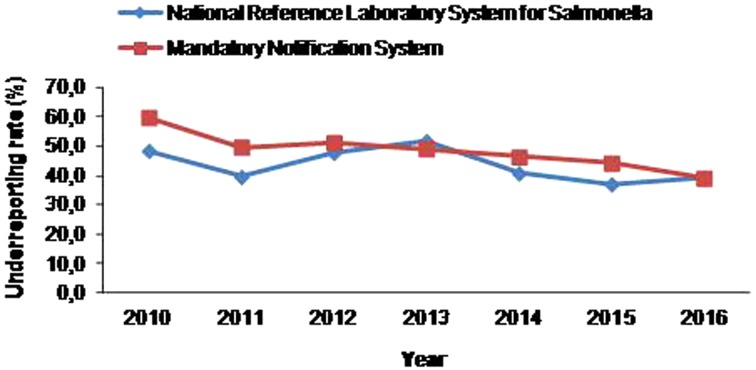
Trend of underreporting rate of salmonellosis at the Mandatory Notification System and the National Reference Laboratory System for *Salmonella* by year, 2010–2016.

Regarding the underreporting rate of salmonellosis to MNS there was a statistically significant decreasing trend (*P* = 0.002) over the period 2010–2016, while in NRLS system no significant trend in underreporting was observed (*P* = 0.212) (Fig. 2). For both notification systems, even though there was no statistically significant seasonal trend observed (MNS: *P* = 0.087, NRLS: *P* = 0.169), the mean annual underreporting rates increased during winter months reaching a peak in December and gradually decreased in summer (Table 3).

### Factors affecting MNS and NRLS notification rates

In MNS, private hospitals and hospitals located in Attica had significantly higher salmonellosis underreporting rates than public and district hospitals (*P* = 0.048 and *P* = 0.025, respectively), while in NRLS system, public, district and small hospitals had significantly higher underreporting rates than private and large hospitals in Attica (*P* = 0.002, *P* = 0.002 and *P* = 0.029, respectively) (Table 3).

### Comparison of the two systems

The two sources were independent since the odds ratio was close to unity; therefore, comparison of the two notification systems was possible. Salmonellosis underreporting of MNS was significantly higher compared with underreporting of NRLS throughout the study period. Also, MNS had statistically significant higher underreporting rates from private hospitals compared with the NRLS system (*P* = 0.001) (Table 4).

**Table 4.** Underreporting rates by year and month of notification, type and size of hospital and geographical region; Mandatory Notification System and National Reference Laboratory System for *Salmonella*, Greece, 2010–2016

	*Salmonella* spp.		
		Underreporting % (Mean rates)	*P*-value
Year of notification	MNS	48.5	0.147
	NRLS	43.5	
Month of notification	MNS	48.1	0.313
	NRLS	44.5	
Type of hospital (public/private)	MNS	51.0	0.001
	NRLS	30.2	
Hospital beds (<250/⩾251)	MNS	44.4	0.894
	NRLS	44.0	
Region (Attica/Rest of Greece)	MNS	46.7	0.243
	NRLS	40.0	

## Discussion

Knowledge of the epidemiology of foodborne diseases is vital for planning, implementing and evaluating public health policies and practices [7, 26]. In order to be able to estimate the actual burden of a disease in the country, data on the laboratory capacity of medical services and underreporting rates to surveillance systems are needed, amongst others [6, 27]. In Greece, there is a lack of published literature regarding the performance of surveillance systems of foodborne diseases, thus the estimation of their actual burden is difficult [9].

Based on the hospital records, besides the financial crisis, laboratory capacity of the Greek hospitals to perform cultures and diagnose salmonellosis was high and steady throughout the study period.

Capture-recapture analysis is a useful and not costly way to estimate the actual number of laboratory-confirmed cases at Greek hospitals, to evaluate salmonellosis underreporting rate with accuracy, to interpret collected data appropriately, to identify weaknesses of surveillance systems and to indicate factors associated with underreporting rate in order to apply correction measures for the improvement of the system's completeness [28, 29].

A well-known limitation of capture-recapture methods is their sensitivity to their underlying four assumptions. Authors believe that assumptions were not seriously violated in this study: (1) the study population was closed, (2) all cases had the same probability of being captured to each of the systems (catchability), (3) cases of the surveillance systems were identified and matched and (4) there was independence between the two sources [23, 24, 30, 31].

According to the results of this capture-recapture study, the actual number of laboratory-confirmed cases at Greek hospitals was substantially higher than that reported from MNS and NRLS system, resulting in an estimated incidence rate almost twice than that reported. Consequently, notification rates substantially underestimate the actual number of laboratory-confirmed salmonellosis cases at Greek hospitals.

Furthermore, the underreporting rate of salmonellosis in Greece decreased during the period 2010–2016 for both surveillance systems in place. Sensitisation initiatives of HCDCP since 2010 seem to have contributed to this decrease. Estimated underreporting of salmonellosis in this study is similar to previous estimations in the country [9, 32, 33] and to estimations of reporting rates in Spain [34]. Also, austerity measures did not seem to have deteriorated the completeness of surveillance systems for salmonellosis even though they might have decreased the degree of potential improvement due to the initiatives of the last years.

On the other hand, underreporting of salmonellosis remains higher than that reported from other European countries (e.g. Sweden, [35], Germany [36], Italy [36], Ireland [37]) depicting that reporting has not been optimal and new strategies are needed.

In Greece, a significant seasonal trend is observed for notified salmonellosis cases, with the mean annual notification rate increasing during summer, reaching a peak in August and gradually decreasing in autumn [5], which is a seasonality pattern consistent with findings from other European countries (Malta, Poland, Portugal, Romania, Slovakia and Spain) [2]. Underreporting rate seems to follow a pattern reciprocal to the seasonality pattern of notification rate. Higher undernotification in winter months, with a peak in December, can be explained by a reduced alertness of the clinicians to notify cases when the disease is less frequent and consequently perceived as not important from a public health point of view [32].

Analysis of underreporting at MNS showed that hospitals located at the capital notify less frequently. This finding may be attributed to the increased workload since almost 50% of the population of the country resides in Attica and also to the constant changing of personnel working at the hospitals in the capital. These findings are in accordance with other studies [6, 37–40], showing that excess work, lack of time and proper training of personnel on disease reporting are associated with increased underreporting rates. On the contrary, analysis of underreporting of NRLS showed that large hospitals located in Attica send isolates to the reference centre more, probably due to the lower transportation cost, compared with hospitals located in other regions. Also, larger hospitals probably have a better mechanism in place in order to cover the cost of transportation to the reference centre than smaller ones.

The fact that estimated underreporting for salmonellosis, in MNS, was statistically significantly higher for private hospitals and that in NRLS system it was higher for public hospitals, might reflect the different manners that public and private hospitals use the different systems; it has been observed that private hospitals make use of the system for providing more information to their clients, while clinicians of public hospitals do not consider the serotype as a clinically relevant information and avoid sending the isolates to the reference lab, especially for sporadic cases.

A striking finding is that the underreporting rate of MNS, which is a compulsory system, is higher than the underreporting rate of NRLS system, which is a voluntary system. This result shows that clinicians working at the hospitals notify less new salmonellosis cases through MNS compared with microbiologists. Inadequate training of clinical doctors on the importance of surveillance systems and on the basic public health concepts and principles may be a possible explanation of this difference, however further investigation of the reasons that clinical doctors do not report as systematically as microbiologists are needed.

Ideally, all cases should be notified and the respective isolates to be sent to the reference laboratory in order to have the full completeness of the two systems. Apparently, based on the results of our study this is not the case and we need to increase the completeness of both systems. In order to have more valid data regarding the actual number of salmonellosis cases diagnosed at Greek hospitals, we can conclude that data from the two systems should be combined. The existence of two or more parallel systems for the surveillance of salmonellosis is common in Europe and allows each system to complement the other while it enables crossover conclusions drawn from the analysis of the information collected by each system separately [35]. Record-linkage has been proved to be an important tool for assessing the quality and completeness of registers and in several European countries, clinical and laboratory notifications are combined in order to meet these objectives [35, 41].

Following the example of other European countries, we recommend that the implementation of a common electronic database for both systems that will allow for each system to systematically feed the other is needed in Greece. In this way, when an isolate is sent to the reference laboratory automatically a mandatory notification form will be required from the clinical doctor with a reminder that notification of the disease is mandatory. Additionally, when a mandatory notification form is sent a reminder for sending the isolate to the reference laboratory with practical guidelines may help in increasing completeness of NRLS, too.

In conclusion, the underreporting rate of confirmed salmonellosis cases decreased during the follow-up period (2010–2016) besides the financial crisis, however underreporting of both systems is still high. Differences in the underreporting rate by geographical region, size and type of hospital can guide interventions for the improvement of notification. Efforts to make clinical doctors more aware of reporting to MNS should be continuous, especially for doctors working at private hospitals located in Attica, while for NRLS, efforts should focus at smaller public hospitals in other regions and transportation cost of isolates should be addressed. Record linkage and use of a common electronic database in order one system to feed the other could significantly increase the completeness of both systems.

Investing in better surveillance systems and public health interventions is a cost-saving approach in austerity situations since it is the only way to early detect public health threats and implement preventive measures and thus reduce the cost for health care services (hospitalisation, medication, etc) [42].

## Author contributions

KM conceived of the study and its design, led the study organisation, coordination, data collection and interpretation and contributed to the data analysis, as well as the manuscript's first draft and revisions. ESP participated in the design of the study, collection and entry of the data, performed the statistical analysis and drafted the manuscript. GM participated in the study design, contributed to the data analysis, interpretation and the review of the manuscript. TG reviewed the background, the methodology, the interpretation and the manuscript. All authors read and approved the final manuscript.
